# Comparing Childhood Characteristics of Adopted and Non-adopted Individuals Deceased by Suicide

**DOI:** 10.3389/fpsyt.2022.756306

**Published:** 2022-06-03

**Authors:** Fabienne Ligier, Festus Body Lawson, Marilou Lamourette, Charles-Edouard Giguère, Alain Lesage, Monique Séguin

**Affiliations:** ^1^McGill Group on Suicide Studies, Montréal, QC, Canada; ^2^Psychiatry Department, Montréal University, Montréal, QC, Canada; ^3^Research Center, Institut Universitaire en Santé Mentale de Montréal, Montréal, QC, Canada; ^4^EA 4360 APEMAC, Université de Lorraine, Nancy, France; ^5^PUPEA, Centre Psychothérapique de Nancy, Laxou, France; ^6^Banque Signature, Research Center, Institut Universitaire en Santé Mentale de Montréal, Montréal, QC, Canada; ^7^Québec Network on Suicide Research, Québec, QC, Canada; ^8^Department of Psychoeducation and Psychology, Québec University, Québec, QC, Canada; ^9^Centre Intégré de Santé et Service Social de l'Outaouais (CISSSO), Gatineau, QC, Canada

**Keywords:** adoption, developmental course, risk factors, suicide, youth—young adults

## Abstract

**Objective:**

Across the globe more than 35,000 children a year are adopted by non-relatives, and some studies suggest that adopted individuals may be more vulnerable to developing mental disorders. To map the differences in suicide risk factors in adopted and non-adopted individuals, this study will compare the development of mental disorders as well as life events occurring before the age of 18 for both adopted and non-adopted individuals deceased by suicide.

**Methods:**

This study included 13 adopted and 26 non-adopted individuals deceased by suicide as well as 26 non-adopted living control individuals. Cases were taken from a data bank created over the last decade by researchers of [our institution] comprising a mixture of 700 suicide cases and living control individuals aged from 14 to 84. Adopted and non-adopted individuals deceased by suicide; adopted individuals deceased by suicide and non-adopted living control individuals were each compared on Axis I and II disorders, early life events, and burdens of adversity.

**Results:**

Results show significant differences, with a higher rate of Attention Deficit Hyperactivity Disorder, mental health comorbidity and Cluster C personality disorders among adopted individuals. Furthermore, adopted individuals have higher adversity scores prior to the age of 15.

**Conclusion:**

This study underlines the fact that adoptive families need to be supported throughout adoption. Health care professionals need specialized training on this matter, and the psychological challenges adopted individuals face need to be treated at the earliest juncture.

## Introduction

Across the globe, more than 35,000 children a year are adopted by non-relatives. Fortunately, the majority of adopted individuals are in good physical and mental health (1, 2). The knowledge that they were given up for adoption, or the experience of adverse early life events may be counteracted by a nurturing family environment (3). Some authors refer to the capacity to overcome early adversity, transforming experiences into resilience, especially when adoptive parents are sensitive to issues relating to the origins of their adopted child (4, 5).

However, when compared to the general population, a significantly large proportion of adopted individuals will develop mental disorders during childhood or later in life, which suggests that adopted individuals may be more prone to developing mental disorders (6–8). Recent data suggests that biological inheritance may be involved in the development of mental disorders among adopted individuals (9, 10); family antecedent of mental disorders accounts for 33–43% of suicide risk in adopted individuals (11, 12) as well as a proportion of mood disorders and substance abuse (9, 10). In addition, early life exposure to institutional deprivation may have a negative effect during the development process, and may increase the presence of mental disorders (13–16). For example, some studies reveal that adopted individuals may have a higher risk of externalizing disorders such as Attention Deficit Hyperactivity Disorder (ADHD) (2, 17), substance abuse (18), as well as a higher risk of suicide attempts (19).

Suicide is the second leading cause of death among people aged 15–24 and suicide prevention is a public health priority (WHO). Since adoption was during several decades an increasing trend worldwide, and adopted individuals may be more vulnerable to suicide, it is important to consider the specific risk variables these individuals may have to bear, and if prevention strategies need to be adapted for this specific population (20).

To map the differences in suicide risk factors in adopted and non-adopted individuals, this study will compare the development of mental disorders as well as life events occurring before the age of 18 for both adopted and non-adopted individuals deceased by suicide.

## Patients and Methods

### Participants and Recruitment

Thanks to an ongoing partnership between (our institution) and the Quebec Coroner's Office, for the past two decades several research groups [Dumais et al. (21); Kim et al. (22); Séguin et al. (23–25)] have been able to document the life trajectories of individuals deceased by suicide by interviewing their bereaved family members (23, 24, 26). The protocol is as follows: the family receives an introductory letter from the coroner's office, then a research assistant follows up with a telephone call. A trained mental health clinician then contacts the family members in order to present the study. If the family members agree to participate in our study an appointment is made, and the interview process begins 3–4 months after the suicide. Two interviews, each approximately 3 h long, are conducted for each suicide case. Approximately 75% of the close relatives referred by the coroner's office agreed to participate in the study.

Control participants were interviewed over the course of several studies. Most control individuals were participants from the general population identified through a snowball sampling method and an informant who had known the control participants were interviewed (23). This procedure has been previously described by Dumais et al. (21) and Kim et al. (22). All participants signed a consent form and the research held REB approval.

Over the last decade, researchers have created a data bank comprising a mixture of 700 suicide cases and control participants, aged 14–84 (23, 24, 26). In this data bank, we identified 13 cases of adopted individuals deceased by suicide which were compared with 26 non-adopted individuals deceased by suicide, and with 26 non-adopted living control individuals.

### Measurements

Data on common sociodemographic characteristics, life events and mental health characteristics were collected.

#### Interview to Determine Post-mortem Diagnosis

The post-mortem diagnosis was assessed using a psychological autopsy method (27). During the interview semi-structured questionnaires were administered using the DSM-IV Structured Clinical Interview for both Axis I and Axis II disorders (SCID I and II) (28), with an informant who had known the deceased well (26). Hospital files were also examined to corroborate this information and determine whether a diagnosis of mental disorder was present.

A case vignette was then drafted and discussed by a panel of experts, to determine the post-mortem diagnosis by consensus. This panel was composed of researchers from our team, clinical practitioners, psychiatrists, and psychologists.

A series of studies over the past decade have established the concordance of DSM diagnoses generated by informant reports in conjunction with chart diagnoses and the psychological autopsy method, which have been proven to have good reliability (27, 29, 30). The same interview methodology was applied to a control group (with a proxy-based interview or direct interview if proxy was unavailable).

#### Interview to Retrace Life Trajectory

The Life Trajectory Calendar interview method was borrowed from Life History Calendar research (30). The questionnaire uses a Life History Calendar to reconstruct the major events in an individual's life as an aid to accurately recall significant life experiences. The calendar explores several clearly described variables from all life spheres; furthermore the frequency, severity and duration of each variable is indicated on the calendar. Narrative methodology requires clinical case histories (case vignettes), and Life History Calendars were drafted after the interviews; the Life History Calendar makes it possible to pinpoint the occurrence of specific events (both positive and negative). The frequency, severity and duration of each event is recorded, and classified in a specific life sphere such as: events associated with early adversity (abuse, neglect, presence of violence, etc.,); events associated with academic life (interruptions, successes, failures, education path etc.); events associated with professional life (unemployment, stress at work, promotions, etc.,); events associated with social life (presence or absence of social support, friends, colleagues, etc.,); events involving the onset of interpersonal difficulties (difficulties associated with mental health, suicide attempts, illness, etc.,). The Life History Calendar approach, as underlined in a previous paper, assists in identifying proximal and distal life events, which helps to understand the life trajectories of individuals deceased by suicide (24). This interesting approach also allows the burden of adversity over the life trajectory to be quantified (24). For this study we targeted the variables occurring before the age of 19 years old.

#### Burden of Adversity

A variable of 5-year periods for measuring the burden of adversity was developed in order to combine events occurring during a specific period of age into a 'summary variable. The value of this global variable identified as the “burden of adversity” was determined by a panel of experts (25). From clinical case histories, the panel analyzed the life trajectories of each individual and gave an overall adversity rating for each five-year period. The overall burden of adversity assessments ranged from severe (rating 1 or 2), to moderate (3 or 4), to low (5 or 6). In all cases, the experts rated each five-year period independently before reaching a consensus through discussion. When studying the clinical case histories, the intra-pair agreement rating in our panel of experts for each five-year period ranged from 76 to 97%; the lowest agreement was found in the 0–4-year age group studied (24).

### Analysis

In the aforementioned data bank, there were 13 adopted individuals among the 305 suicide cases. Each adopted individual deceased by suicide was matched (1:2) with a non-adopted individual deceased by suicide by age, gender, and region of study (New Brunswick, Ontario, or Québec) at the moment of death. Each adopted individual was also matched with a non-adopted living control individual (1:2) by the same variables (also matched by age, gender and region of study, at the time of interview for the control individuals).

Analyses were made by comparing Axis I and II disorders, early life events, and the burdens of adversity between adopted and non-adopted individuals deceased by suicide, and between adopted individuals deceased by suicide and non-adopted living control individuals. Axis I disorders were distinguished by 2 periods: the 12 months prior to death (individuals deceased by suicide) or the 12 months prior to the interview (living individuals) and the period preceding the12 months prior to the suicide or interview. We also compared the age of suicide of deceased adopted and non-adopted individuals. Comparisons were made using the Chi Square and Student's *t*-test, with *p* < 0.05 for significance. Analyses were carried out with SAS 9.4 software.

## Results

This study included 65 individual cases: 13 adopted individuals deceased by suicide, 26 non-adopted individuals deceased by suicide, and 26 non-adopted living control individuals. Each group was 54% male.

The mean (*SD*) age at the time of adoption was 10 months (18), while the mean age of suicide for both adopted and non-adopted individuals (*n* = 39) was 33.8 (19.6), from 13 to 83.

### Adopted vs. Non-adopted Individuals Deceased by Suicide

Comparisons of Axis I or II disorders between adopted and non-adopted individuals deceased by suicide show no difference for Axis I diagnoses in the 12 month period prior to death (Table 1). For the period preceding the 12 months prior to death, Attention Deficit Hyperactivity Disorder (*p* < 0.0001) and having two or more Axis I diagnoses (*p* = 0.004) are over-represented among adopted individuals. The same can be said for for Axis II Cluster C personality disorders (*p* = 0.04).

**Table 1:** Comparison of an Axis I and II disorders in the 12 months prior to suicide, and the period preceding the 12 months prior to suicide between adopted and non-adopted individuals (*n* = 39).

**Characteristics**	**Adopted individuals deceased by suicide (*n =* 13)**	**Non-adopted individuals deceased by suicide (*n =* 26)**	**Chi2 value**	***p-*value**
**12 months prior to suicide**	
Mood disorder	9	15	0.17	0.68
Substance abuse and dependence disorder	6	8	0.89	0.34
Psychosis/schizophrenia	0	2	1.07	0.29
Adjustment disorder	1	4	0.46	0.49
Eating disorder	1	1	0.25	0.62
Two or more disorders	6	10	0.21	0.64
Total with only one DX	4	11	0.49	0.49
**Period preceding the 12 months prior to suicide**	
Substance abuse and dependence disorder	7	7	2.72	0.10
Mood disorder	5	6	1	0.32
Attention Deficit Hyperactivity Disorder	3	0	6.5	0.01^*^
Anxiety disorder	2	1	1.62	0.23
Gambling	1	3	0.13	0.72
Eating disorder	1	1	0.25	0.63
Psychosis/schizophrenia	0	1	0.5	0.48
Total with only one DX	5	10	0	1
Two or more disorders	7	3	8.15	0.004^*^
**Axis II disorder**	
Cluster A	1	0	2.09	0.15
Cluster B	6	6	2.16	0.14
Cluster C	5	3	3.84	0.04^*^

* *significant value with Chi2 test*.

As for life events occuring prior to the age of 19 (Table 2), there was no difference between groups.

**Table 2:** Childhood life-events comparison between adopted and non-adopted individuals deceased by suicide (*n* = 39).

**Characteristics**	**Adopted individuals deceased by suicide (*n =* 13)**	**Non-adopted individuals deceased by suicide (*n =* 26)**	*p* ^*^
**Age 0–4**	
Discipline/neglect/tensions in the parent-child relationship	9	11	0.11
Sexual/physical abuse	5	6	0.31
**Age 5–9**	
Discipline/neglect/tensions in the parent-child relationship	8	12	0.36
Sexual/physical abuse	3	4	0.55
Academic difficulties	1	3	0.71
**Age 10–14**	
Discipline/neglect/tensions in the parent-child relationship	7	13	0.82
Mental health problems	8	8	0.08
Sexual/physical abuse	5	6	0.31
Academic difficulties	2	4	1
Substance abuse	1	3	0.71
**Age 15–19**	
Discipline/neglect/tensions in the parent-child relationship	9	17	0.81
Mental health problems	7	12	0.39
Sexual/physical abuse	4	8	1
Academic difficulties	3	2	0.13

* *p-value with Chi2 test*.

**Figure 1:**
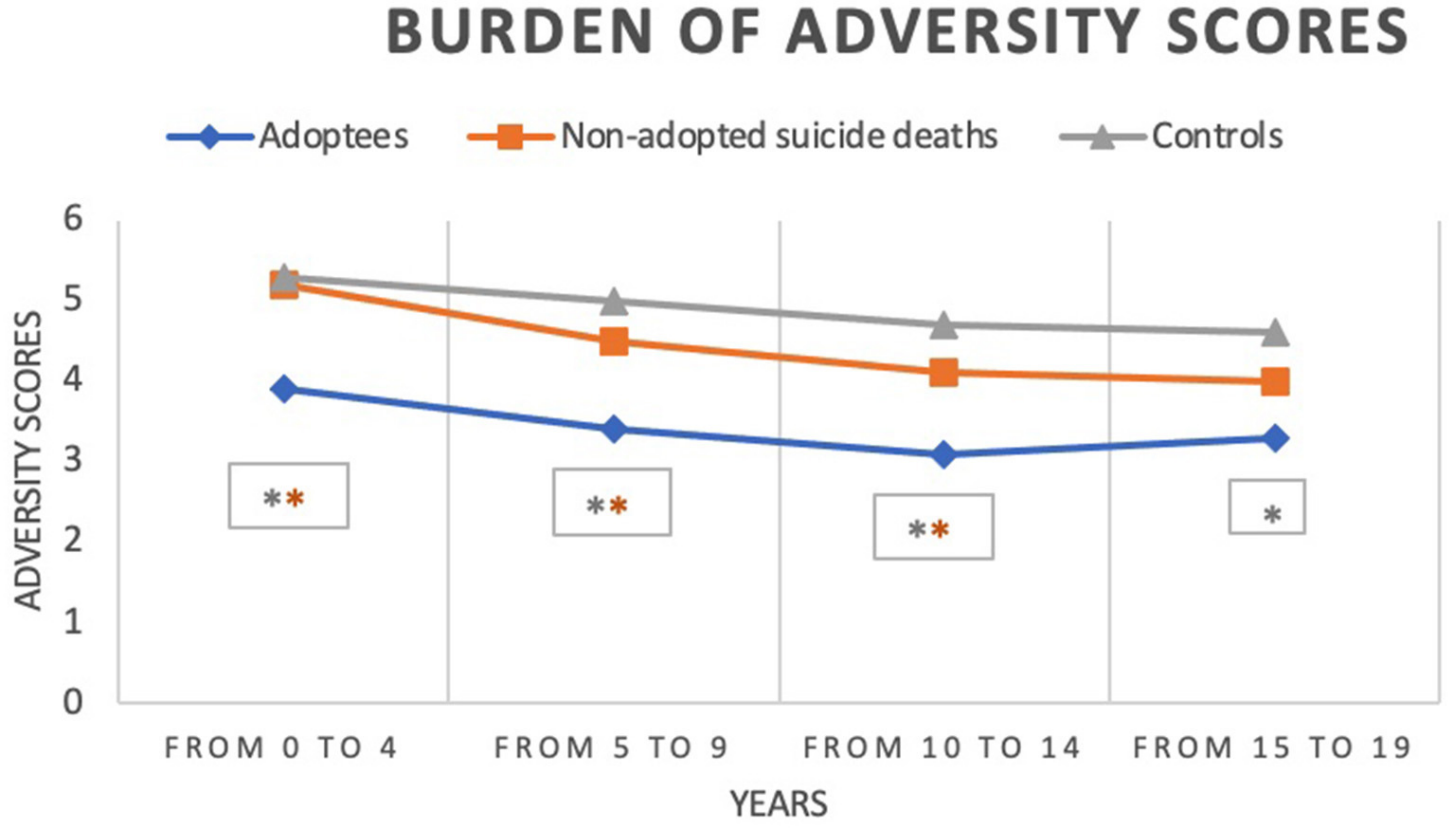
Burden of adversity scores comparing adopted and non-adopted individuals deceased by suicide, and non-adopted living control individuals (*n* = 65). *p < 0.05. The orange line compares adopted and non-adopted individuals deceased by suicide, and the silver line compares adopted and control individuals. The lower the score number, the higher the burden of adversity.

However, adopted individuals deceased by suicide have moderate adversity scores compared with the low adversity scores measured in non-adopted individuals deceased by suicide (see Table 3, Figure 1). The difference in adversity scoring is significant: from age 0 to 4 (3.9 vs. 5.3, *p* = 0.003), from 5 to 9 years old (3.4 vs. 5.0, *p* < 0.0001) and from age 10–14 (3.1 vs. 4.7, *p* < 0.003). The difference is however non-significant in the 15–19 age bracket (*p* = 0.14).

**Table 3:** Comparison of the burden of adversity score depending on age between adopted and non-adopted individuals deceased by suicide (*n* = 39).

**Adversity score *the lower the score number, the higher the burden***	**Adopted individuals deceased by suicide (*n =* 13) Mean (SD)**	**Non-adopted individuals deceased by suicide (*n =* 26) mean (SD)**	***p***
Age 0–4	3.9 (1.8)	5.2 (1.1)	0.01^*^
Age 5–9	3.4 (1.1)	4.5 (2.3)	0.009^*^
Age 10–14	3.1 (2.4)	4.1 (1.3)	0.02^*^
Age 14–19	3.3 (0.9)	4.0 (1.5)	0.14

* *significant value with Student test*.

Lastly, there is no significant difference in the presence and number of past suicide attempts in adopted and non-adopted individuals deceased by suicide (0.07 < *p* < 0.49).

### Adopted Individuals Deceased by Suicide vs. Control Individuals

Comparisons of Axis I or II disorders between adopted individuals deceased by suicide vs. control individuals show significant differences in mood disorders (*p* < 0.0001), substance abuse (*p* = 0.04) and the presence of two or more Axis I diagnoses (*p* = 0.0002). These characteristics are over-represented among adopted individuals deceased by suicide. There is no difference for Axis I diagnoses in the period preceding the 12-months prior to their death or interview (0.06 < *p* < 1). There are significant differences for Axis II: adopted individuals deceased by suicide have higher incidences of Cluster B and C personality disorders compared with control individuals (*p* = 0.005 for both).

When looking at life events prior to the age of 19, adopted individuals deceased by suicide have higher rates of the variable “discipline/neglect/tensions in parent-child-relationship” (*p* = 0.04) and more mental health problems from the age of 10 years old (*p* = 0.02) compared with non-adopted living control individuals.

Finally, adversity scores are all significantly higher for individuals deceased by suicide with *p* < 0.003 for each period (see Figure 1).

## Discussion

The aim of this study was to compare adopted with non-adopted individuals deceased by suicide to find a potential specificity in adopted individuals deceased by suicide. Results show significant differences: a higher incidence of ADHD, mental health comorbidity and Cluster C personality disorders among adopted individuals. Moreover, adopted individuals have higher adversity scores prior to the age of 15.

Adopted individuals cumulate two or more Axis I diagnosis in the period preceding the 12 months prior to death, including ADHD. According to the literature, ADHD diagnosis is significantly higher in adopted individuals (17), which may be explained by several factors. One of these factors may be immaturity of the mother: giving up one's baby for adoption may be associated with teenage pregnancy and Halmøy and colleagues (31) concluded from a large population-based study, that adults with ADHD were more likely to be firstborns and to have a younger maternal age at delivery. Another factor may be mental disorders and/or substance abuse, as well as alcohol or drug absorption *in utero* (32, 33). These factors may in fact be consequences of a history of ADHD in the parents which increases the risk of ADHD in the adopted child (34–36). Equally, if we focus on parental substance abuse, infants may suffer withdrawal symptoms, which is associated with a higher risk of anxiety, a trait shared in the personality disorders found in the adopted individuals examined in this study. Indeed, personality disorders included in Cluster C of Axis II are Avoidant, Dependent, and Obsessive-Compulsive Personality Disorders. The weight of heredity may have both direct and indirect impact in the development of mental disorders among adopted individuals and may partly explain the cumulation of Axis I diagnoses.

Aside from the heredity factor, the early trauma of abandonment in adopted individuals may disturb the quality of attachment and their relationship with adoptive parents. Attachment disorders may also be explained by excessive expectations from the parents that lead to feelings of disappointment. Among the individuals in this study, Cluster B personality disorders were diagnosed in 6 of the 13 adopted individuals. We may hypothesize that, perhaps unsurprisingly, due to early separation from their biological parents some of the individuals studied may have been traumatized at an early stage in life, and may have therefore developed an attachment disorder (1), a characteristic often associated with Cluster B personality disorders such as borderline personality disorder.

If we focus on suicide risk factors, anxiety and borderline personality disorders are both well-known suicide risk factors, as is the impulsivity found in ADHD diagnosis (23, 37). So adopted individuals cumulate Axis I and Axis II mental health disorders for several reasons, and these diagnoses are all associated with a higher risk of suicide.

Furthermore, in all individuals deceased by suicide, mental health problems appear early: between 10 and 14 years old. The mean age of suicide is approximately 33, which underlines that suicide prevention strategies must be embedded from childhood. Of course, family relationships play a buffering role to protect family members against some risk factors, including the risk of suicide and we do not study here the quality of adoptive parents and their child (38).

Even if adopted and non-adopted individuals deceased by suicide have the same types of adverse early life events, adopted individuals have an increased early life adversity burden, even if they were adopted in their first year of the life. May we therefore hypothesize that some adopted individuals were less resilient, and so less able to overcome their early-life trauma (5)?

If we summarize the “profile” of adopted individuals deceased by suicide: they have a greater combination of psychiatric comorbidities and they have higher adversity scores in comparison with non-adopted individuals deceased by suicide compared with the control group. In addition, they have a higher rate of mood disorder and substance abuse during the 12 months prior to suicide compared with individuals from the general population deceased by suicide. They also have higher rate of Cluster B and C personality disorders, as usually described in their life trajectory (23).

That is why it is crucial to be attentive to adopted individuals, a population that may present higher hereditary risk factors for mental disorders, as underlined in the literature (9, 10). The recommendation for special attention may be also linked to this question: do adopted individuals have more psychiatric diagnoses because their parents are more attentive to their psychological development, as described by some authors (8)? Would this explain why adopted individuals often have more diagnoses? In this study, diagnoses have been made in the same way for adopted and non-adopted individuals through interviews of their informants, so the results should have not be influenced by the fact of been or not been adopted. But in situations in which the individual has been adopted, professionals run the risk of trivializing symptoms in saying “it”s just due to their being adopted', as perhaps may occur when dealing with their anxiety issues. We know adopted individuals need care, at least as much if not more than than their non-adopted peers: professionals must therefore be trained specifically to take into account an adopted individual's background when providing care.

This study has some limitations due to the retrospective methods employed, specifically regarding memory biases. However, a series of studies over the past decade have established agreement between DSM diagnoses based on informant report and those based on medical charts (39) and have shown the psychological autopsy method to be reliable (29, 40, 41). As is common in this kind of study, the control individuals are not representative of the general population, as they were generally recruited from friends or neighbors who share environmental and associative mating determinants of mental disorders, which may explain the high rate of mental disorder among control individuals (42). At last, we have no information about adoption between national and international. However, the fact that this is an original study is a strength, as this kind of research has not been done on suicide subjects before, plus we matched individuals on gender, age and region of study to limit biases.

## Conclusion

Adopted individuals who die by suicide have higher adversity scores in early life, even if they were adopted in their first year of life. Besides the potential trauma of abandonment, they may have hereditary risk factors for mental disorders. Youth caregivers have to be carefull to these risks of suicide in general and in adopted children in particular.

The adoptive family need to be supported throughout adoption, health care professionals need specific training, and psychological difficulties need to be cared for at the earliest possible juncture.

## Data Availability Statement

The raw data supporting the conclusions of this article will be made available by the authors, without undue reservation.

## Ethics Statement

The studies involving human participants were reviewed and approved by Research Ethics Boards of the Douglas Mental Health Institute (Montreal) and of the Université du Québec en Outaouais. The patients/participants provided their written informed consent to participate in this study.

## Author Contributions

FL and MS conceptualized and designed the study, drafted the initial manuscript, and reviewed and revised the manuscript. C-EG, FL, and AL designed the data collection instruments, collected data, carried out the initial analysis, and reviewed and revised the manuscript. FB and ML critically reviewed the manuscript for important intellectual content. All authors approved the final manuscript as submitted and agree to be accountable for all aspects of the work.

## Conflict of Interest

The authors declare that the research was conducted in the absence of any commercial or financial relationships that could be construed as a potential conflict of interest.

## Publisher's Note

All claims expressed in this article are solely those of the authors and do not necessarily represent those of their affiliated organizations, or those of the publisher, the editors and the reviewers. Any product that may be evaluated in this article, or claim that may be made by its manufacturer, is not guaranteed or endorsed by the publisher.

## Abbreviations

ADHD: Attention Deficit Hyperactivity Disorder.
